# Cimicosis in Persons Previously Fed Upon by Bed Bugs

**DOI:** 10.7759/cureus.5941

**Published:** 2019-10-18

**Authors:** Johnathan M Sheele, Cameron Crandall, Brandon F Chang, Brianna L Arko, Colin Dunn, Alejandro Negrete

**Affiliations:** 1 Emergency Medicine, Mayo Clinic, Jacksonville, USA; 2 Emergency Medicine, University Hospitals, Cleveland, USA; 3 Clinical Research / Trauma Surgery, The MetroHealth System, Cleveland, USA

**Keywords:** bedbug, bed bug, cimex lectularius, survey, rash, pruritic, pruritis, blister, prevalence, emergency department

## Abstract

Introduction

Bed bug infestations have risen dramatically in many industrialized nations in recent decades. Most people fed upon by bed bugs will develop a pruritic rash although the frequency with which this occurs is not definitively known and may depend on host factors including the duration of the infestation.

Methods

Surveys were completed from 706 emergency department (ED) patients in Cleveland, OH about their current and past exposure with bed bugs. Subjects were asked about any post-bed bug feeding rashes that developed.

Results

There were 24% (169/698) of subjects reporting either a current or past home bed bug infestation, with 37% (253/698) reporting they had previously been fed upon by a bed bug. Of those reporting a previous bed bug feeding, 68% (172/253) reported a pruritic post-bed bug feeding rash and 24% (57/237) reported developing a blister. Overall, 5% (37/705) of ED patients reported currently having a rash, but only 2% (14/698) of ED patients reported currently have bed bugs at home and of those, only 14% (2/14) said they currently had a rash.

Conclusion

While 68% of ED patients reported a pruritic post-bed bug feeding pruritic rash, almost a third of persons did not report developing the rash. Post-bed bug feeding blister reactions are less common. Asking ED patients about a rash had a low sensitivity of 14% (2-43%) and a specificity 95% (93-96%) to identify persons reporting home bed bugs.

## Introduction

The common bed bug, *Cimex lectularius* L., is an obligate hematophagous insect that preferentially feeds on humans. Bed bugs can be one of the most common ectoparasites that clinicians encounter in industrialized nations. Within the hospital, bed bugs have been found mostly in the emergency department (ED) [1]. One hospital reported finding a bed bug within the institution every 2.2 days and within the ED every 4-5 days, resulting in a significant institutional financial burden [1-3]. ED patients with bed bugs were more likely to be older, male, arrive to the ED by ambulance, and admitted to the hospital [4-6].

Most people fed on by *C. lectularius* develop cimicosis, which typically manifests as a nonspecific pruritic maculopapular rash, but which can also present with bullae, vesicles, or resemble a vasculitis [7]. The prevalence of cimicosis in persons with bed bug infestations is unknown, and there are no reports on the frequency of cimicosis in hospitalized patients with bed bugs [7-8]. Cimicosis typically develops in a previously unexposed person about 10 days after the initial feeding. In sensitized individuals, the time from bed bug exposure to a reaction can be almost immediate [8]. Summaries of dermal reactions from bed bug feedings have been previously published [7-13].

The purpose of our study was to assess the frequency of self-reported pruritic and blistering cimicosis in patients reporting they had previous been fed upon by bed bugs. Additionally, we sought to determine the frequency of self-reported rashes in ED patients and assess whether this correlates with having a home bed bug infestation.

## Materials and methods

We received institutional review board (IRB) approval by University Hospitals to survey ED patients at a single, tertiary care, academic center in downtown Cleveland, Ohio, between June and October 2017. We surveyed 706 ED patients ≥18 years of age who did not have psychosis, homicidal ideation, altered mentation, or dementia. Data was collected seven days a week, predominantly during day and evening hours, and we surveyed ~2-3% of all ED patients during that time period. Patients reporting “unsure” or had “no answer” as responses were not included in the final data analysis. For continuous variables the mean and standard deviation (SD) were reported and analyzed using the independent t-test or analysis of variance (ANOVA). Categorical variables were summarized by frequency or percentage and analyzed using Chi-square. Binomial logistic regressions were performed using the presence of home bed bug infestations as the dependent variable. An alpha of 0.05 was set for statistical significance.

## Results

Two percent of ED patients (14/698) reported having a current home bed bug infestation, and 24% (169/698) of patients reported having either a current or past home bed bug infestation. Those with a current bed bug infestation were significantly older, 52 years (standard deviation (SD) 14; n = 14) compared to those without bed bugs, 41 years (SD 18; n = 684) (p = 0.02), respectively (Table 1) [5-6]. Regression analysis for patients reporting a current bed bug infestation and controlling for homelessness in the past year, education level, and annual income showed that age remained a significant predictor for having a current infestation (Odds ratio (OR) 1.04; 95% confidence interval (CI) 0.009 to 0.068 [p = 0.01]). Reporting a current home bed bug infestation was not associated with currently having a rash in the ED, insomnia, homelessness in the last year, annual income, or education level on univariate analysis (p > 0.05) (Table 1) [5-6].

**Table 1:** Patients reporting a current home bed bug infestation. RR = relative risk; 95% CI = 95% confidence intervals; SD = standard deviation; ED = emergency department

	+ bed bugs (n = 14)	-bed bugs (n = 684)	RR (95% CI)	P value
Mean age in years (SD)	52 (14)	41 (18)	(-20.5 to 1.90)	0.02
Current rash in the ED			1.11 (0.89 to 1.37)	0.13
Yes	14% (2/14)	5% (35/683)		
No	86% (12/14)	95% (648/683)		

Thirty-seven percent of patients (253/680) reported a past history of being fed upon by a bed bug. Binomial logistic regression for ever having been fed upon by a bed bug with age as a covariate found an Odds ratio of 0.99 (95% CI -0.01 to 0.029) (p = 0.02). However, when also controlling for homelessness in the past year, education level, and annual income, age was not a significant predictor (OR 0.99; 95% CI -0.02 to 0.002 [p = 0.14]). Persons reporting previously being fed upon by a bed bug (mean: 39 years (SD 16; n = 253)) were younger than those who had never been fed upon by a bed bug (mean: 42 years (SD 18; n = 427)) (p = 0.02) (Table 2). This suggests that older patients are more likely to report a home bed bug infestation but younger patients were more likely to report having been fed upon by a bed bug. Lastly, there were significant differences between patients reporting a previous bed bug feeding and those who reported homelessness in the past year 56% (24/43) compared to no homelessness 36% (226/630) (p = 0.009). Having a current rash in the ED was not associated with ever having been fed upon by a bed bug (p = 0.36). Sixty-nine percent (175/253) of patients reported their last bed bug feeding was ≥12 months ago (Table 2).

**Table 2:** Patients reporting they had previously been fed upon by a bed bug. RR = relative risk; 95% CI = 95% confidence interval; SD = standard deviation

	Yes (n = 253)	No (n = 427)	RR (95% CI)	P
Mean age in years (SD)	39 (16)	42 (18)	(0.56 to 5.99)	0.02
Homelessness in last year			1.45 (1.03 to 2.04)	0.009
Yes	56% (24/43)	44% (19/43)		
No	36% (226/630)	64% (404/630)		
Current rash in the ED			1.02 (0.98 to 1.06)	0.36
Yes	6% (16/253)	5% (20/426)		
No	94% (237/253)	95% (406/426)		
Pruritic cimicosis	68% (171/235)	32% (82/235)		
Mean age (SD)	39 (16)	39 (16)	(-4.00 to 4.55)	0.90
Blistering cimicosis	24% (57/237)	76% (180/237)		
Mean age (SD)	41 (15)	38 (16)	(-7.47 to 2.03)	0.26
Time from last bed bug feeding		NA	NA	NA
≤2 months	8% (20/253)			
2-6 months	14% (35/253)			
6-12 months	9% (22/253)			
≥12 months	69% (175/253)			
Annual income			NA	<0.001
	71% (170/240)	52% (212/406)		
$25,000-$50,000	25% (59/240)	28% (114/406)		
$50,000-$75,000	3% (6/240)	9% (38/406)		
$75,000-$100,000	1% (3/240)	4% (17/406)		
≥$100,000	1% (2/240)	6% (25/406)		
Education level			NA	<0.001
≤ high school	90% (226/252)	76% (323/426)		
Associate’s	8% (19/252)	9% (38/426)		
Bachelor’s	2% (4/252)	10% (44/426)		
Master’s	1% (3/252)	3% (14/426)		
Doctorate	0% (0/252)	2% (7/426)		

Pruritic cimicosis

Sixty-eight percent of patients (172/253) reporting a past bed bug feeding stated they developed a post-feeding dermal reaction that was pruritic. There were no significant differences (p > 0.05) in those patients that reported pruritic cimicosis versus no pruritis cimicosis and annual income, insomnia, having a current rash in the ED, homelessness in the last year, the time from their last known bed bug feeding, and having a current home bed bug infestation on univariate analysis. Additionally, binomial logistic regression for pruritic cimicosis when adjusting for annual income and homelessness in the last year found that age was not a significant predictor for pruritic cimicosis (OR 1.004; 95% CI 0.99 to 1.02 [p = 0.64]).

Blistering cimicosis

Twenty-four percent (57/237) of patients with a previous report of a bed bug feeding developed a blistering rash. Eighty-six percent (49/57) of persons reporting a previous bed bug feeding reported the bed bug feeding resulted in both pruritic and blistering cimicosis versus 14% (8/57) that reported blistering cimicosis without pruritis. There were no significant differences (p > 0.05) in those patients that reported a blistering versus non-blistering cimicosis and annual income, insomnia, homelessness in the last year, and the time from the last bed bug feeding. Binomial logistic regression for the development of blistering cimicosis when adjusting for annual income and homelessness in the last year found that age was not a significant predictor for pruritic cimicosis (OR 1.02; 95% CI 1.0 to 1.04 [p = 0.13]).

Current rash in the emergency department

Overall, 5% (37/705) of ED patients reported currently having a rash while in the ED. There were no significant differences for patients with and without a rash in the ED and age, level of formal education, annual income, reported insomnia, or homelessness in the past year on univariate analysis (p > 0.05). Those patients that reported having a rash in the ED were asked to rank the degree of pruritis they were having on a scale of 1-5 (with 1 meaning no itch and 5 meaning extreme pruritis). The mean degree of pruritis was 2.9 (SD 1.6; n = 34).

Current rash in the emergency department and bed bugs

On univariate analysis those patients in the ED reporting a current rash were not significantly more likely to also report a home bed bug infestation (14% (2/14)) compared to those without bed bugs (5% (35/682)) (p = 0.13); however, the low number of patients with bed bugs affects the generalizability of these findings. Using the presence of a current rash in the ED to screen for a home bed bug infestation had a sensitivity of 14% (2-43%), specificity 95% (93-96%), positive predictive value 5% (2-18%), and negative predictive value 98% (98-99%). Having a current home bed bug infestation was not associated with having a current rash in the ED using logistic regression and controlling for age, homelessness in the past year, education level, annual income (OR 1.01; 95% CI 0.99 to 1.03 [p = 0.28]). Interestingly, 21% (3/14) of patients with bed bugs at home reported never having been fed on by a bed bug.

## Discussion

Older age was a significant predictor for having a current bed bug infestation even when controlling for potential confounding variables. On univariate analysis younger age was significantly associated with a higher chance of reporting ever having been fed upon by a bed bug, but this association was no longer significant when controlling for potential confounders. Homelessness in the past year was associated with a significant increased risk for reporting ever having been fed upon by a bed bug.

Most people reporting a previous bed bug feeding report that they developed pruritis (68%), and fewer, reported developing a blister (24%). Our findings are similar to those of a survey of persons with bed bugs which found that 74% developed a skin reaction to being fed up on by the insect [9]. Blistering cimicosis has been reported previously in the setting of a severe reaction to the bed bug feeding although the frequency of blistering cimicosis in the general public is unknown [14].

Current and previous exposure to bed bugs can be common among ED patients with 37% in our survey reporting that they have previously been fed upon by a bed bug. Patients with bed bugs may not be forthcoming about their infestations with their healthcare providers [5]. Bed bugs are small and seek harborage away from their human host when they are not feeding, making it harder for healthcare providers to identify an insect during a clinical encounter [2]. Diagnosing bed bug infestations can be even more challenging without a pruritic rash as was reported in 32% of subjects in our survey. Only 14% (2/14) ED patients reporting an active home bed bug infestation also reported having a rash in the ED, compared with 5% with a rash in the overall ED patient population. Using the presence of a current rash in the ED as a triage screening question for having an active home bed bug infestation had a low sensitivity (14%) but high specificity (95%), and therefore would not likely be helpful despite the high burden of bed bugs within this population.

Limitations

There are no clinical diagnostic tests for current or past exposure to bed bugs, and we were unable to confirm the presence of any bed bug exposures in our subjects. Some subjects may have felt that they were fed upon by bed bugs when in fact it may have been a different insect. Lastly, subjects may not have been able to accurately recall their past reactions to bed bugs especially since a majority of subjects reported their last bed bug feeding was ≥ 12 months ago.

## Conclusions

While a majority of ED patients reported a pruritic post-bed bug feeding rash, it is notable that almost a third of persons did not report developing a pruritic rash. Twenty-four percent of persons reported developing a post-bed bug feeding blister. The number of ED patients reporting a rash was more than twice the number that reported currently having a home bed bug infestation. Most ED patients with a home bed bug infestation did not report having a rash in the ED. Asking ED patients if they currently have a rash as a method of screening them for a reported home bed bug infestation has a low sensitivity. Results suggest that older patients are more likely to report a home bed bug infestation but younger patients were more likely to report having been fed upon by a bed bug.
